# *Spiroplasma eriocheiris* Invasion Into *Macrobrachium rosenbergii* Hemocytes Is Mediated by Pathogen Enolase and Host Lipopolysaccharide and β-1, 3-Glucan Binding Protein

**DOI:** 10.3389/fimmu.2019.01852

**Published:** 2019-08-08

**Authors:** Mingxiao Ning, Yunji Xiu, Meijun Yuan, Jingxiu Bi, Libo Hou, Wei Gu, Wen Wang, Qingguo Meng

**Affiliations:** ^1^Jiangsu Key Laboratory for Aquatic Crustacean Diseases, College of Marine Science and Engineering, Nanjing Normal University, Nanjing, China; ^2^College of Life Sciences, Nanjing Normal University, Nanjing, China; ^3^Marine Science and Engineering College, Qingdao Agricultural University, Qingdao, China; ^4^Co-innovation Center for Marine Bio-Industry Technology of Jiangsu Province, Lianyungang, China

**Keywords:** *Macrobrachium rosenbergii*, *Spiroplasma eriocheiris*, interactive proteins, innate immunity, infection

## Abstract

*Spiroplasma eriocheiris* is a crustacean pathogen, without a cell wall, that causes enormous economic loss. *Macrobrachium rosenbergii* hemocytes are the major targets during *S. eriocheiris* infection. As wall-less bacteria, *S. eriocheiris*, its membrane protein should interact with host membrane protein directly and firstly when invaded in host cell. In this investigation, six potential hemocyte receptor proteins were identified firstly that mediate interaction between *S. eriocheiris* and *M. rosenbergii*. Among these proteins, lipopolysaccharide and β-1, 3-glucan binding protein (MrLGBP) demonstrated to bind to *S. eriocheiris* using bacterial binding assays and confocal microscopy. Four spiroplasma ligand proteins for MrLGBP were isolated and identified. But, competitive assessment demonstrated that only enolase of *S. eriocheiris* (SeEnolase) could be a candidate ligand for MrLGBP. Subsequently, the interaction between MrLGBP and SeEnolase was confirmed by co-immunoprecipitation and co-localization *in vitro*. After the interaction between MrLGBP and SeEnolase was inhibited by antibody neutralization test, the virulence ability of *S. eriocheiris* was effectively reduced. The quantity of *S. eriocheiris* decreased in *Drosophila* S2 cells after overexpression of *MrLGBP*, compared with the controls. In addition, RNA interference (RNAi) knockdown of *MrLGBP* made *M. rosenbergii* more sensitive to *S. eriocheiris* infection. Further studies found that the immune genes, including *MrLGBP* and *prophenoloxidase* (*MrproPO*), *MrRab7A*, and *Mrintegrin* α*1* were significantly up-regulated by SeEnolase stimulation. After SeEnolase pre-stimulation, the ability of *M. rosenbergii* resistance to *S. eriocheiris* was significantly improved. Collectively, this investigation demonstrated that MrLGBP and pathogen SeEnolase involved in mediating *S. eriocheiris* invasion into *M. rosenbergii* hemocytes.

## Introduction

Host-pathogen contact is a prerequisite for bacterial invasion and colonization (1, 2). Spiroplasma, a wall-less bacterium (3), undoubtedly depends upon protein interactions between the bacterium and cells of the host. As a novel spiroplasma, *Spiroplasma eriocheiris* is the causative agent of tremor-disease (4). Previous investigations have demonstrated *Macrobrachium rosenbergii* hemocytes to be the main cellular targets of *S. eriocheiris*, from which infections disseminate into the prawn body (5). It is likely that proteins mediate the interaction between *S. eriocheiris* and hemocytes, permitting entry of spiroplasma into hemocytes.

Lipopolysaccharide and β-1, 3-glucan binding protein (LGBP), a pattern recognition protein (PRP), recognizes and binds to common epitopes on the pathogen surface (6). Subsequent to recognition (7), LGBP activates distinctly a series of immune responses, including phagocytosis, nodule formation, clotting cascade, the synthesis of a wide array of antimicrobial peptides, and the prophenoloxidase system (proPO) (8–10). Our previous research (11) has shown that in *M. rosenbergii* hemocytes infected with *S. eriocheiris*, 69 differentially expressed proteins including LGBP were identified compared with control group injected R2 medium [8% sucrose, 2.5% heart infusion broth (HIB), 15% fetal bovine serum (FBS), 100 U ml^−1^ penicillin, and pH 7.20–7.40]. These results suggested that LGBP play an important role in pathogen invasion into host. However, the specific mechanism by which LGBP facilitates *S. eriocheiris* entry into *M. rosenbergii* has not been elucidated.

In order to bind and penetrate target cells, attachment organelles very likely contain specialized receptors similar to those of human mycoplasmas (12, 13). Several spiroplasma adhesins have been identified. These include adhesin-like protein (ALP) (14) and spiralin (15), both of which are involved in spiroplasma transmission. *Spiroplasma citri* colonization of insect cells is promoted by the interaction of phosphoglycerate kinase (PGK) with actin (16). Further, enolase, a key cytoplasmic glycolytic enzyme, is found on the cell surface of *Mycoplasma fermentans* (17) as well as *Streptococcus pneumoniae* (18). Enolase and its receptor protein, plasminogen, are known to promote bacterial binding to host cells. However, no investigations have identification proteins that mediate the interaction of *S. eriocheiris* with *M. rosenbergii* hemocytes. Herein, identification of bacterial-host interaction proteins that play a complex and important role in the process of *S. eriocheiris* entry into *M. rosenbergii* hemocytes.

## Materials and Methods

### Spiroplasma Strain, Freshwater Prawns, and Primary Hemocyte Culture

*Spiroplasma eriocheiris*, obtained from a naturally infected *M. rosenbergii*, was derived from a livestock farm in Gaoyou, Jiangsu province of China (5) and cultured in R2 liquid medium (8% sucrose, 2.5% HIB, 15% FBS, 100 U ml^−1^ penicillin, and pH 7.20–7.40) at 30°C (4).

Healthy *M. rosenbergii* freshwater prawns were obtained from a commercial farm in Nanjing, Jiangsu province of China, and reared in tanks at 28°C with freshwater and an aeration system. A polymerase chain reaction (PCR) was conducted to guarantee that the prawns were free of spiroplasma (19). Prawns were fed daily for 2 weeks with a commercial diet before hemocytes were withdrawn.

*Macrobrachium rosenbergii* primary hemocytes (20) were cultured at 28°C in Leibovitz-15 (L-15) growth medium (pH 7.2–7.4) supplemented with 15% FBS, 0.1% glucose, 0.5% NaCl, and antibiotics (100 U ml^−1^ penicillin, 100 U ml^−1^ streptomycin, and 1 μg ml^−1^ amphotericin b).

### Identification of Receptor Proteins

This experiment was conducted based on the methods of Labroussaa et al. (21), with modifications. Hemocytes were withdrawn from the second abdominal segment of healthy *M. rosenbergii* using a 1 ml sterile syringe containing 500 μL modified phosphate buffer saline (PBS) (0.9 g/L Na_2_HPO_4_, 0.27 g/L KH_2_PO_4_, 0.6 g/L KCl, 25.5 g/L NaCl, and 1.0 g/L glucose, pH 7.2) as anticoagulant. The diluted hemocytes were centrifuged for 5 min at 3,800 × g to collect cells and resuspended in Common Lysis Buffer (Generay, China) containing 1 mM phenylmethanesulfonyl fluoride (PMSF). After the mixture was centrifuged for 3 min at 10,000 × g, a bicinchoninic acid (BCA) procedure was used to assess protein concentration. Aliquots of supernatant (20 μg) were separated by electrophoresis in a 12.5% sodium dodecyl sulfate polyacrylamide gel electrophoresis (SDS-PAGE) gel, and then transferred from the gel to a polyvinylidene difluoride (PVDF) membrane. After transfer, membrane was blocked in 10 ml of tris-buffered saline with Tween (TBST) with 10% bovine serum albumin (BSA) overnight at 4°C. Then, membranes were incubated with 10 ml TBST containing 5% BSA and 5 μg/ml formaldehyde-killed spiroplasma (22) at room temperature (RT) for 1 h. For the control experiment, spiroplasma were not included. After incubation, membrane was washed three times with TBST and incubated in 10 ml of TBST containing 5% BSA with purified polyclonal antibodies (0.5 μg/ml) reactive with *S. eriocheiris* at a dilution of 1:2,000 at RT for 1 h. After three washings with TBST, membrane was incubated with peroxidase-conjugated goat anti-rabbit IgGs (Transgen Biotech, China) at a 1:20,000 dilution in 5% BSA at RT for 1 h. Detection of the bands was by incubation with an enhanced chemiluminescence (ECL) substrate solution (E411-01/02) according to the manufacturer's instructions (Vazyme Biotech, China). Membranes was washed three times with TBST and then exposed to X-ray film. Based on the method of Killiny et al. (23), proteins of interest (11) were excised from stained gels and digested with trypsin. Peptide mass spectrometry (MS) and tandem mass spectrometry (MS/MS) were performed by BIO-TECH (China) using an ABI 5800 MALDI-TOF/TOF Plus mass spectrometer (Applied Biosystems, USA). Proteins of interest were successfully identified at 95% or higher confidence using the MASCOT V2.3 search engine (Matrix Science Ltd., London, U.K.).

### Binding of Recombinant Proteins to Spiroplasma

A pair of primers, MrLGBP-F/-R (Table S1), were used to amplify a 1101-bp open reading frame (ORF) encoding a mature protein. PCR fragments were digested with *Bam*H I and *Not* I restriction enzymes and cloned into a pGEX-4T1 plasmid. The recombinant plasmids pGEX-4T1-MrLGBP were transformed into *Escherichia coli* Transetta (DE3) cells for isopropyl-b-D-thio-galactoside (IPTG) (final IPTG concentration of 0.5 mM) induced glutathione S-transferase (GST)-tagged recombinant expression. MrLGBP was purified by Glutathione Sepharose 4 Fast Flow (GE Healthcare).

The bacteria binding assay was conducted as described previously (24). Briefly, spiroplasma were cultured overnight in R2 medium and during exponential growth, collected by centrifugation at 9,000 × g for 5 min at RT. Bacteria were washed three times with PBS and thoroughly resuspended in PBS to an OD_600_ of 1.0. The purified recombinant MrLGBP was incubated with 100 μL of *S. eriocheiris* (2 × 10^8^ cells/ml) at RT with gentle rotation for 2 h, washed four times with PBS, and spiroplasma eluted with 100 μL of 8 M urea at RT for 30 min. After centrifugation at 9,000 × g for 5 min, supernatant and precipitate were loaded onto a 12.5% SDS-PAGE gel. A western blotting experiment using anti-GST monoclonal antibodies was conducted to detect the direct binding of recombinant proteins to spiroplasma. Bacteria cells were subjected to the same treatment and incubated with the GST tag as a control.

### Co-location of MrLGBP and Spiroplasma by Confocal Microscopy

Primary *M. rosenbergii* hemocytes cultures were established based on the method of Du et al. (20). After 5 h of seeding, the hemocytes were infected with 100 μL of spiroplasma (10^8^ cells/ml) for 16 h at 28°C. Unbound bacteria were removed by washing three times with PBS. Hemocytes were immersed in fixative (4% paraformaldehyde in PBS) for 30 min at RT. Fixed hemocytes were rinsed three times with PBS and permeabilized with PBS containing 0.5% Triton X-100 for 10 min at RT, and then incubated in blocking buffer (PBS plus 5% BSA) at RT for 1 h.

For MrLGBP and spiroplasma co-localization, hemocytes were incubated at RT for 1 h with rabbit anti-*S. eriocheiris* (diluted 1:5,000) and mouse anti-MrLGBP (diluted 1:4,000) polyclonal antibodies (prepared by Vazyme Biotech, China) in PBS containing 1% BSA. After three washings, the nuclei was stained with 4′,6-diamidino-2-phenylindole, dihydrochloride (DAPI) and MrLGBP was stained by Alexa Flour 555 donkey anti-mouse IgGs (Beyotime, China) at a 1:7,000 dilution. Spiroplasma was stained at RT for 1 h by Alexa Flour 488 goat anti-rabbit IgGs (Beyotime, China) at a 1:10,000 dilution in PBS containing 1% BSA. After washing, immunofluorescent samples were visualized with a confocal laser scanning microscope (Nikon TI-E-A1R, Japan).

### Identification of Ligand Proteins

*Spiroplasma eriocheiris* was collected by centrifugation (14,000 × g, 10 min) and then washed three times with PBS. Washed spiroplasma was suspended in Common Lysis Buffer [50 mM Tris (pH 7.4), 150 mM NaCl, 1% NP-40, 1 mM PMSF, and 2 mM ethylene diamine tetraacetic acid (EDTA)] and treated at 50% duty cycles and an intensity of 400 W for 20 min at 4°C. After the mixture was centrifuged for 5 min at 10,000 × g, the supernatant was fractionated by SDS-PAGE, and then transferred from the gel to a PVDF membrane. After blocking, membrane was incubated for 2 h with purified recombinant MrLGBP or the GST tag at a final concentration of 2 μg/ml in TBST containing 5% BSA. The protocol was similar to that described in the “Identification of Receptor Proteins” section except that anti-GST monoclonal antibody at a dilution of 1:2,000 (Transgen Biotech, China) was used instead of spiroplasma-reactive polyclonal antibody.

### Competitive Assay of SeEnolase in Hemocyte

In the “Identification of Ligand Proteins” assay, SeEnolase, transketolase (TK), and acetaldehyde dehydrogenase (ALDH) were successfully identified. To determine whether ligand proteins participate in the infection process of *S. eriocheiris*, the gene coding for ligand proteins were amplified and cloned into a pEASY-BluntE1 expression vector (Transgen Biotech, China). Since, TGA is read as a tryptophan codon and not as a termination signal in most *Mollicute* species, the TGA codons were mutated to TGG codons by the Fast Mutagenesis System (Transgen Biotech, China) according to the instructions. Amplification and mutagenesis primers are listed in Table S1. The recombinant plasmids were transformed into *E. coli* Transetta (DE3) for protein expression. Recombinant proteins were purified via Ni Sepharose 6 Fast Flow (GE Healthcare). Samples were analyzed by SDS-PAGE.

Primary *M. rosenbergii* hemocytes were incubated with different ligand proteins at a final concentration of 20 μg/ml, and infected by adding 10 μL of spiroplasma (10^8^ cells/ml) for 16 h at 28°C. Uninfected cells served as positive control, and cells incubated with BSA as a negative control. Following incubation, the hemocytes were then washed three times with PBS to remove spiroplasma that were not attached. Cell viability was determined with cell counting kit-8 (CCK-8) reagent (Beyotime, China) using 10 μL/well for 2 h, and measured the optical density (OD) at 450 nm using a microplate reader (25). To maintain consistency, all data were reported as relative cell viability as the mean ± S.E. Statistical significance was determined by one-way analysis of variance (ANOVA) and by *post-hoc* Duncan multiple range tests.

### Localization of SeEnolase

According to our previous research methods (26), the rabbit poly-antibody of SeEnolase was successfully prepared, namely anti-SeEnolase serum. The proteins from *S. eriocheiris* and purified recombinant SeEnolase were analyzed by western blot using the anti-SeEnolase serum.

Cytoplasm proteins and outer membrane proteins from *S. eriocheiris* were obtained as previously described (27, 28), with modification. Briefly, spiroplasma was collected by centrifugation at 12,000 × g for 20 min, washed three times, and resuspended in Tris-HCl (0.02 mol/L, pH 7.5) followed by sonication in an ultrasonic disintegrator (200 W). The sonicate was ultra-centrifuged at 34,000 × g for 30 min, and the supernatant (cytoplasmic proteins) and the pellet (membrane proteins) were collected. Membrane proteins were re-suspended in PBS with a proteinase inhibitor. Protein samples were fractionated by electrophoresis by SDS-PAGE. For western blotting, anti-SeEnolase serum (anti-adhesin serum and anti-arginine deiminase serum were used as controls) was used as the primary antibody (1:2,000) and goat anti-rabbit IgG (whole-molecule) peroxidase conjugate (Sigma) as the secondary antibody (1:5,000). Blots were developed with a 3,3′,5,5′-Tetramethylbenzidine Liquid MB Substrate Kit (Promega, USA).

### Antibody Neutralization Assay

An antibody neutralization test was used to investigate the effect of anti-SeEnolase serum on *S. eriocheiris* infection of *M. rosenbergii*. The bacteria were pretreated by incubating with anti-SeEnolase serum, pre-immune serum, and PBS, respectively, for 1 h at 30°C (29). Healthy prawns (average 4–5 g, *n* = 50 for each group) were randomly divided into three groups, anti-SeEnolase serum + *S. eriocheiris*, pre-immune serum + *S. eriocheiris*, PBS + *S. eriocheiris* group. In the anti-SeEnolase serum + *S. eriocheiris* group, 20 μL pretreated bacteria with anti-SeEnolase serum were injected into prawns. Meanwhile, 20 μL of pretreated bacteria with pre-immune serum or PBS were injected for the pre-immune serum + *S. eriocheiris* or PBS + *S. eriocheiris* group. Five prawns were randomly selected from three groups for analysis of *S. eriocheiris* copies at 1, 3, 5, 7, and 9 days, respectively. The number of *S. eriocheiris* copies in *M. rosenbergii* hemocytes were determined by a real-time PCR using primers Se-QF and Se-QR (Table S1) (30).

To further test prawn mortality, 50 μL of pretreated bacteria with anti-SeEnolase serum, pre-immune serum, or PBS were injected for the anti-SeEnolase serum + *S. eriocheiris*, pre-immune serum + *S. eriocheiris*, or PBS + *S. eriocheiris* group. At the same time, three another groups were injected with the anti-SeEnolase serum, pre-immune serum, and PBS, respectively. The cumulative mortality of prawns was recorded daily.

### Co-immunoprecipitation and Co-localization Assay

To generate pAc-enolase-V5 and pAc-enolase-RFP plasmids for expression of V5-tagged and RFP-tagged SeEnolase protein, the ORF of SeEnolase was cloned into pAc5.1-V5 and pAc5.1-RFP vectors (31, 32) at *Kpn* I and *Apa* I sites using the gene-specific primers pAc-enolase-F and pAc-enolase-R (Table S1). Similarly, ORF of MrLGBP was cloned into pAc5.1-GFP vectors using the primers pAc-MrLGBP-F and pAc-MrLGBP-R (Table S1).

For the co-immunoprecipitation assay, pAc-enolase-V5 was co-transfected with pAc-MrLGBP-GFP or pAc5.1-GFP (as a control) into *Drosophila* S2 cells. Forty-eight hours after transfection, cells were harvested and washed with ice-cold PBS three times, and then lysed with NP-40 lysis buffer (Beyotime, China) with 1 mM PMSF protease inhibitor (Solarbio, China), and incubated with 2 μg of anti-GFP mouse antibody (Transgen Biotech, China) or anti-V5 mouse antibody (Transgen Biotech, China) overnight at 4°C. Antibodies were precipitated with 40 μL of protein G resin beads (Transgen Biotech, China) for 3 h at 4°C. Beads were then washed three times in ice-cold NP-40 lysis buffer and samples subjected to SDS-PAGE. Western blotting was performed with anti-GFP rabbit antibody (1:2,000) or anti-V5 rabbit antibody (1:2,000), and peroxidase-conjugated goat anti-rabbit secondary antibody at a dilution of 1:5,000 (Transgen Biotech, China). A standardized aliquot (10%) of each total input cell lysate was also examined as a control.

*Drosophila* S2 cells were seeded onto cover-glass bottom dishes with ~60% confluence and then co-transfected with 2 μg pAc-enolase-RFP and 2 μg pAc-MrLGBP-GFP using the FuGENE HD Transfection Reagent (Promega, USA). At 48 h post-transfection, following three ice-cold washes with PBS, cells were incubated with DAPI (Beyotime, China) for 10 min at RT. The fluorescent images were visualized with a confocal laser scanning microscope (Nikon TI-E-A1R, Japan).

Methods of culture, immobilization, permeability, and blocking of primary *M. rosenbergii* hemocytes have been described in the section “Co-location of MrLGBP and Spiroplasma by Confocal Microscopy.” Then, hemocytes were incubated with 1 ml PBS containing 1% BSA and 2.5 μg/ml SeEnolase protein at RT for 1 h. For the control experiment, SeEnolase protein was not included. After three washings, hemocytes were incubated at RT for 1 h with rabbit anti-SeEnolase (diluted 1:1,000) and mouse anti-MrLGBP (diluted 1:4,000) polyclonal antibodies (prepared by Vazyme Biotech, China) in PBS containing 1% BSA. Hemocytes nuclei was stained with DAPI. MrLGBP was stained by Alexa Flour 488 goat anti-Mouse IgGs (Beyotime, China) at a 1:7,000 dilution. SeEnolase were stained at RT for 1 h by PE-labeled Goat Anti-Rabbit IgG (Transgen Biotech, China) at a 1:5,000 dilution in PBS containing 1% BSA. After washing, immunofluorescent samples were visualized with a confocal laser scanning microscope (Nikon TI-E-A1R, Japan).

### Assay for Over-expression of MrLGBP

Using the FuGENE HD Transfection Reagent (Promega, USA), *Drosophila* S2 cells from each dish were transfected with 2 μg pAc5.1-MrLGBP-GFP and pAc5.1-GFP plasmids, respectively. Twenty-four hours later, the *Drosophila* S2 cells were infected with *S. eriocheiris* (10^8^ cells/ml). *Drosophila* S2 cells were divided into three groups, *S. eriocheiris* only, *S. eriocheiris* + GFP, and *S. eriocheiris* + LGBP-GFP. At 48 h, after *S. eriocheiris* infection, samples were washed three times with PBS and fixed with Immunol Staining Fix Solution (Beyotime, China). Fixed *Drosophila* S2 cells were rinsed three times with PBS and permeabilized with PBS containing 0.2% TritonX-100 for 30 min, and then incubated in blocking buffer (PBS plus 3% BSA) for 30 min. The cells were incubated with *S. eriocheiris* polyclonal antibody with 1% BSA in PBS overnight. Then, the cells were incubated with PE-labeled Goat anti-Rabbit IgG (Transgen Biotech, China) and examined using a confocal laser scanning microscope (Nikon TI-E-A1R, Japan). The methods for transfection and infection of *Drosophila* S2 cells were as described above. To quantify the copy number of *S. eriocheiris, Drosophila* S2 cells were collected from cell culture dishes from each treatment group at 48 h after *S. eriocheiris* infection and subjected to real-time PCR using the primers Se-QF and Se-QR (Table S1) (30). To confirm MrLGBP over-expression, total protein was extracted with lysis buffer (Beyotime, China) containing 1 mM PMSF and 2 mM EDTA on ice. After sonication and centrifugation (13,000 × g, 15 min, 4°C), the supernatants were collected for protein concentration measurement using the bicinchoninic acid assay (BCA). Thirty micrograms of protein was analyzed using 12% SDS-PAGE and western blotting using anti-GFP (Transgen Biotech, China) and HRP-conjugated Goat Anti-Mouse IgG (Transgen Biotech, China). The bands were visualized using ECL (Vazyme, China).

*Drosophila* S2 cells were seeded into a 96-well plate with a final ~60% confluence. The methods for transfection of *Drosophila* S2 cells were as described above. *Drosophila* S2 cells were divided into four groups, R2 medium, *S. eriocheiris* only, *S. eriocheiris* + GFP, and *S. eriocheiris* + LGBP-GFP. At 48 h after *S. eriocheiris* infection, the viability of *Drosophila* S2 cells from 12 wells for each treatment was determined by CCK-8 according to the manufacturer's instructions. All experiments were performed in triplicate.

### RNA Interference Assay

Using an *in vitro* transcription T7 kit for dsRNA synthesis (Takara, Japan), double-stranded RNAs (dsRNAs) targeting the *MrLGBP* and GFP (as control) genes were synthesized. The DNA template for the *MrLGBP* dsRNA preparation was generated by PCR using the gene-specific primers dsRNA-MrLGBP-F and dsRNA-MrLGBP-R (Table S1). For preparation of GFP dsRNA, the primers dsRNA-GFP-F and dsRNA-GFP-R were used (Table S1).

To investigate the RNA interference (RNAi) efficiency, healthy *M. rosenbergii* were cultured in two groups at room temperature. The prawn injected with 20 μg *MrLGBP* dsRNA as the experimental group and 20 μg GFP dsRNA as the control group, respectively, at the second abdominal segment. After 24 h, the prawns were injected again with the same amount dsRNA. Five prawns were prepared and analyzed by Semi-quantitative PCR using primer pairs MrLGBP-qF/MrLGBP-qR (Table S1) at 48, 72, and 96 h after dsRNA injection, respectively.

The phenol oxidase (PO) activity of hemocytes was determined after *MrLGBP* silencing. Hemocytes were withdrawn from the ventral sinus of experimental prawns at 48, 72, and 96 h, respectively, after the first dsRNA injection. Protein concentration was measured using a Total protein quantitative assay kit (Nanjing Jiancheng, China). Hemocyte PO activity was detected using L-3,4-dihydroxyphenylalanine (L-dopa) dissolved in water according to Liu et al. (33). Briefly, 2 μg of total hemolymph proteins in 435 μL of Tris-HCl (10 mM, pH 8.0) were mixed with 65 μL of freshly prepared L-dopa (3 mg/ml in water). After incubation at room temperature for 30 min, PO activity was measured by monitoring the absorbance at 490 nm and recorded as OD490 per μg total protein.

For pathogen challenge tests, healthy prawns (average 4–5 g, *n* = 50 for each group) were cultured at RT and randomly divided into three groups, PBS + *S. eriocheiris*, dsRNA-GFP + *S. eriocheiris*, and dsRNA-LGBP + *S. eriocheiris*. In the PBS + *S. eriocheiris* group, the prawns were treated with 20 μL of PBS. Meanwhile, 20 μg of GFP dsRNA or *MrLGBP* dsRNA were injected for the dsRNA-GFP + *S. eriocheiris* group, or dsRNA-LGBP + *S. eriocheiris* group. All of the prawns were received an injection of 10 μL *S. eriocheiris* (10^8^ cells/ml). Five prawns were randomly selected from three groups for analysis of *S. eriocheiris* copies at 1, 3, 5, 7, and 9 days, respectively. The number of *S. eriocheiris* copies in *M. rosenbergii* hemocytes was determined by a real-time PCR using primers Se-QF and Se-QR (Table S1) (30). All samples were assessed three times.

The healthy prawns (average 4–5 g, *n* = 30 for each group) were cultured at RT and randomly divided into six groups; PBS, dsRNA-GFP, dsRNA-LGBP, PBS + *S. eriocheiris*, dsRNA-GFP + *S. eriocheiris*, and dsRNA-LGBP + *S. eriocheiris*. The prawns of the dsRNA-LGBP group and dsRNA-LGBP + *S. eriocheiris* group were injected individually with 20 μg of *MrLGBP* dsRNA. The prawns of the dsRNA-GFP group and the dsRNA-GFP + *S. eriocheiris* group were injected individually with 20 μg of GFP dsRNA. The prawns of the PBS group and PBS + *S. eriocheiris* group were injected individually with 20 μL PBS. After 24 h, the prawns were injected again with the same amount dsRNA or PBS. Forty-eight hours after the first injection, the prawns of the PBS + *S. eriocheiris* group, dsRNA-GFP + *S. eriocheiris* group, and dsRNA-LGBP + *S. eriocheiris* group received an injection of 50 μL *S. eriocheiris* (10^8^ cells/ml). The cumulative mortality of prawns was recorded daily.

### SeEnolase Stimulation Assay

One hundred healthy prawns (average 4–5 g, *n* = 50 for each group) were randomly divided into two groups. Fifty prawns were injected individually with 50 μL SeEnolase protein (1 μg/μL) as an experimental group. For the control group, fifty prawns were injected with 50 μL PBS. The hemocytes were sampled from every five individuals at 0, 2, 4, 6, 8, 12, 24, 36, and 48 h post-injection. After extraction, total RNA was reverse-transcribed into cDNA with a PrimeScript RT reagent Kit (Takara, Japan). Quantitative real-time PCR (qRT-PCR) was conducted using a 2 × SYBR Premix Ex Taq Kit (Takara, Japan). GAPDH was amplified for internal standardization using the primers GAPDH-qF and GAPDH-qR (Table S1). The PCR reaction was performed in a 10 μL volume with a SYBR Premix Ex Taq™ Kit (Takara, Japan), 0.4 μM of each specific primer (Table S1), and 1 μL of cDNA in StepOnePlus™ Real-Time PCR System. The relative expression levels of immune relative genes, including *MrLGBP, MrproPO, MrRab7A*, and *Mrintegrin* α*1* were calculated according to the 2^−ΔΔ*CT*^ method. All experiments were performed in triplicate. To maintain consistency, all data are given in terms of relative mRNA expression levels as the mean ± S.E. Statistical significance was determined by Student's *t*-test.

Healthy prawns (average 4–5 g, *n* = 50 for each group) were cultured at RT and randomly divided into two groups, SeEnolase + *S. eriocheiris* and PBS + *S. eriocheiris* group. Fifty prawns were injected individually with 50 μL of SeEnolase protein (1 μg/μL) in SeEnolase + *S. eriocheiris* group. For PBS + *S. eriocheiris* group, fifty prawns were injected with 50 μL of PBS. After 12 h stimulation, all of the prawns were received an injection of 10 μL *S. eriocheiris* (10^8^ cells/ml). The mothed of *S. eriocheiris* copies analysis was described in the section Antibody Neutralization Assay.

To further test prawn mortality, two groups of prawns were received an injection of 50 μL *S. eriocheiris* (10^8^ cells/ml) under the same culture conditions and protein stimulation as above. At the same time, two another groups were injected with the SeEnolase and PBS, respectively. The cumulative mortality of prawns was recorded daily.

## Results

### Identification of Receptor Proteins on *M. rosenbergii* Hemocytes

**Figure 1:**
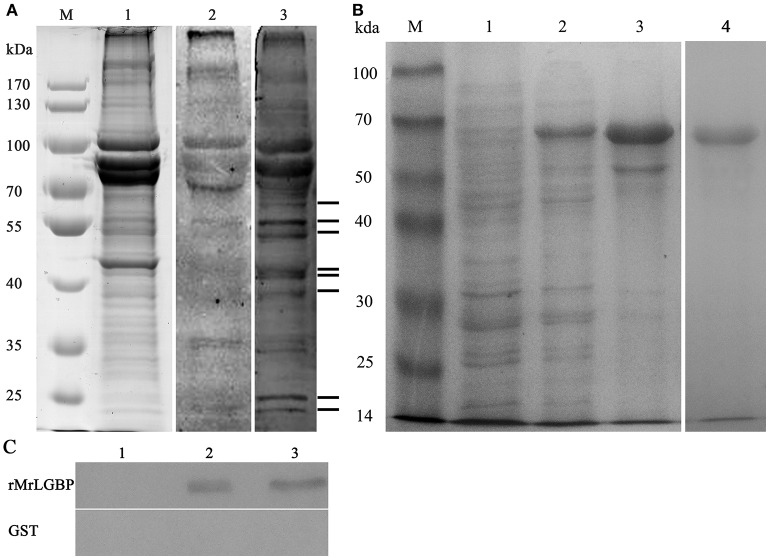
The identified receptor proteins from interactions between *M. rosenbergii* hemocytes and *S. eriocheiris*. **(A)** The far western blotting analysis to identify proteins on hemocytes that could bind to *S. eriocheiris*. *M. rosenbergii* hemocytes total proteins were analyzed by SDS-PAGE (Lane M and 1). Hemocyte receptor proteins were detected by far western blotting (Lane 2 and 3). Lane M, molecular weight marker. Lane 1, *M. rosenbergii* hemocyte proteins (20 μg). Lane 2, control blot not probed with *S. eriocheiris* bacteria. Lane 3, experimental blot probed with *S. eriocheiris* bacteria. Black lines on the right indicate the eight significant binding activities. **(B)** MrLGBP recombinant expression in *E. coli* analyzed by SDS-PAGE. Lane M, molecular weight marker; lane 1, negative control for MrLGBP (without induction); lane 2, supernatant component after induction by IPTG; lane 3, precipitation component after induction by IPTG; lane 4, purified recombinant MrLGBP protein. **(C)** direct binding of MrLGBP to spiroplasma using bacteria binding assay through western blot analysis. GST tag was used as a negative control. Lane 1, eluent of the fourth PBS washing; lane 2, supernatant component after treatment with 8 M urea; lane 3, precipitation component after treatment with 8 M urea.

Far western blotting was used to detect the proteins interacting with *S. eriocheiris*. Compared to the control group, eight different bands were found in the experimental group, having apparent molecular masses of 24, 26, 41, 42, 45, 50, 52, and 56 kDa (Figure 1A). All bands were successfully sequenced, except for the 26 kDa band. By use of blastp and the national center for biotechnology information (NCBI) non-redundant protein database, mass spectra matched the following, ras-related nuclear protein (Ran), MrLGBP, beta-Actin, prophenoloxidase (proPO), beta tubulin, and alpha-tubulin (Figure S1). Related outputs obtained by MASCOT are shown in Table S2.

### Receptor Protein Expression and Spiroplasma Binding Assay

Recombinant MrLGBP proteins were isolated from supernatants after IPTG induction. Apparent molecular weight was 68 kDa by glutathione Sepharose 4B chromatography. After expression and purification, a clear band was detected by SDS-PAGE (Figure 1B, lanes 4). A direct binding assay suggested that although treated with 8 M urea, MrLGBP bound spiroplasma *in vitro* (Figure 1C).

**Figure 2:**
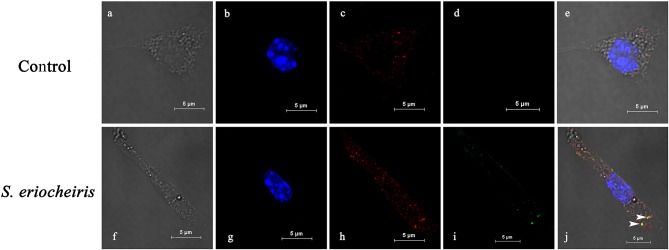
*Spiroplasma eriocheiris* bind to the MrLGBP protein of *M. rosenbergii* hemocyte. The co-localization of MrLGBP and *S. eriocheiris* in prawn hemocytes was analyzed by confocal microscopy. Hemocytes were immobilized after *S. eriocheiris* injection for 16 h. Spiroplasma were stained by Alexa Flour 488 goat anti-rabbit IgGs (green). MrLGBP were stained used the mouse anti-MrLGBP as primary antibody and donkey anti-mouse IgG Alexa-555 as the second antibody (red). Nuclei were stained with DAPI (blue). Hemocytes were visualized by bright field **(a,f)**, blue fluorescence only **(b,g)**, red fluorescence only **(c,h)**, green fluorescence only **(d,i)**, and by bright field merge with all fluorescent molecules **(e,j)** in both the non-infected and *S. eriocheiris* infected group. The white arrows indicate co-localization of MrLGBP and *S. eriocheiris*. Bars = 5 μm.

As shown in Figure 2 of co-localization results, hemocytes were visualized in bright field (Figures 2a,f), blue fluorescence only (Figures 2b,g), red fluorescence only (Figures 2c,h), green fluorescence only (Figures 2d,i), and bright field merge with all fluorescent molecules (Figures 2e,j), respectively. MrLGBP proteins were visualized located in the membrane and cytoplasm (Figures 2c,e). But, *S. eriocheiris* mainly remained on the hemocyte membrane, but a few of them entered the cytoplasm (Figure 2i) after infection for 16 h. So, the co-localization of MrLGBP and *S. eriocheiris* was mainly shown on the membrane, as shown in Figure 2j. These results suggested that *S. eriocheiris* could bind to MrLGBP.

### Identification and Expression of Ligand Proteins of MrLGBP

**Figure 3:**
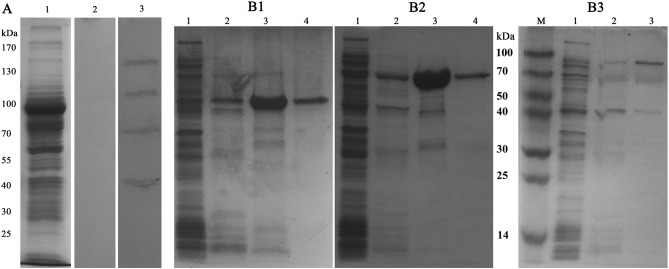
Identification and expression of ligand proteins for MrLGBP. **(A)** Far western blotting was used to identify proteins on *S. eriocheiris* that could bind to MrLGBP. *S. eriocheiris* total proteins were analyzed by SDS-PAGE (Lane 1). *S. eriocheiris* ligand proteins were detected by far western blotting (Lane 2 and 3). Line 1, *S. eriocheiris* proteins (20 μg). Line 2, GST tag as a control blot. Line 3, identification of ligand proteins for MrLGBP. **(B1–B3)** ligand proteins (SeEnolase, TK, and ALDH) recombinant expression in *E. coli* analyzed by SDS-PAGE. Lane 1, flow-through component; lane 2, eluate with 50 mM imidazole; lane 3, eluate with 100 mM imidazole; lane 4, eluate with 500 mM imidazole.

To determine the *S. eriocheiris* ligands interacting with MrLGBP, spiroplasma proteins were fractionated by SDS-PAGE (Figure 3A) and incubated with MrLGBP. The ligand proteins were identified by western blotting using anti-GST monoclonal antibodies. As shown in Figure 3A, lane 3, four significant protein bands, located at ~50, 70, 100, and 130 kDa were found to bind MrLGBP. The corresponding protein bands were excised from colloidal blue stained gels and used for MS/MS analysis. All four proteins were successfully sequenced and blasted. The 50 kDa protein was identified as enolase (GenBank accession number: AHF58090), and the 70 kDa protein were identified as transketolase (TK) (GenBank accession number: AHF57705). The 100 kDa protein was identified as ALDH (GenBank accession number: AHF57596), and the 130 kDa protein was identified as DNA-directed RNA polymerase subunit beta (Figure S2). Related outputs obtained by MASCOT are shown in Table S3. Based on previous reports (18, 34, 35), enolase, TK, and ALDH were focused. Enolase, TK and ALDH of *S. eriocheiris* have five, eight, and five TGA codons, respectively (Figure S3). For expression in *E. coli*, the TGA codons were mutated to TGG using the Fast Mutagenesis System. SeEnolase, TK, and ALDH proteins were successfully expressed and purified (Figures 3B1–B3, respectively).

### Influence of Ligand Proteins on Hemocyte Viability and Localization of *S. eriocheiris* SeEnolase

**Figure 4:**
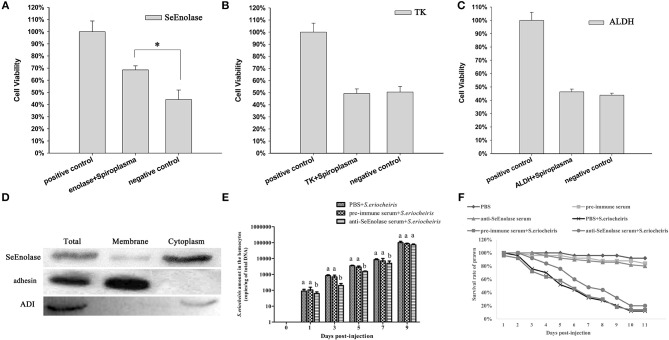
SeEnolase proteins mediate *S. eriocheiris* invasion into *M. rosenbergii* hemocytes. **(A–C)** Show competitive binding experiments with SeEnolase, TK, and ALDH, respectively. *M. rosenbergii* hemocyte were incubated with different ligand proteins and infected by spiroplasma. Cell viability was determined with CCK-8. Cells used for different treatments are shown on the abscissa, and relative cell viability rate on the ordinate. Vertical bars depict the mean ± S.E (*n* = 36). Significant differences were analyzed using one-way ANOVA by *post-hoc* Duncan multiple range tests. *p < 0.05. **(D)** Subcellular localization of SeEnolase. Western blotting was used to analyze the location of SeEnolase, three lanes are represented: total protein, membrane proteins, and cytoplasmic proteins of *S. eriocheiris*, respectively. **(E)** The quantification of *S. eriocheiris* copies in hemocytes from the three groups detected by real-time PCR at 1, 3, 5, 7, and 9 days, respectively. Differences between each group were analyzed using one-way ANOVA. Different letters indicate statistical significance (*p* < 0.05) and the same letter indicate no statistical difference (*p* > 0.05). **(F)** The survival rate of prawn infected using *S. eriocheiris* pretreated with antibody. Pretreated bacteria with anti-SeEnolase serum, pre-immune serum, or PBS were injected into prawn for the anti-SeEnolase serum + *S. eriocheiris*, pre-immune serum + *S. eriocheiris*, or PBS + *S. eriocheiris* group. Three another groups were injected with the anti-SeEnolase serum, pre-immune serum, and PBS, respectively. The cumulative mortality of prawns was recorded daily.

Once *S. eriocheiris* enolase, TK and ALDH were demonstrated to be involved in adhesion to prawn hemocyte surfaces, hemocyte surface proteins were identified by competitive binding. Hemocyte viability percentages were determined after infection with *S. eriocheiris*. The results showed that with SeEnolase incubation (Figure 4A), relative cell viability increased 20% (*p* < 0.05) compared with the negative control. However, for TK and ALDH, there was little or no effect on relative cell viability (Figures 4B,C).

Western blot analysis of proteins from *S. eriocheiris* and purified recombinant SeEnolase using the anti-SeEnolase serum suggested that a protein of 50 kDa was detected (Figure S4). No immunoreactive band was detected in the control group using pre-immune rabbit serum. As shown in Figure 4D, SeEnolase was detected in lanes containing *S. eriocheiris* total protein, membrane proteins, and cytoplasmic proteins, suggesting that SeEnolase is exposed on the surface of *S. eriocheiris*. *S. eriocheiris* adhesin and arginine deiminase (ADI) were previously demonstrated (11) to be *S. eriocheiris* membrane and cytoplasmic proteins, respectively, as controls.

### Enolase Antibody Prevented *S. eriocheiris* Invasion Into *M. rosenbergii* Hemocytes

As shown in Figure 4E, the copies of *S. eriocheiris* in the pre-immune serum + *S. eriocheiris* or PBS + *S. eriocheiris* group was significantly increased in the hemocytes from 1 to 7 days compared to the anti-SeEnolase serum + *S. eriocheiris* group (*p* < 0.05). These results shown that anti-SeEnolase serum could effectively prevent the *S. eriocheiris* invasion into *M. rosenbergii*. And, the survival rate of the pre-immune serum + *S. eriocheiris* or PBS + *S. eriocheiris* group was decreased compared with the anti-SeEnolase serum + *S. eriocheiris* group (Figure 4F). The number of live prawns during this experiment was recorded in Table S4. The results showed that the neutralization of SeEnolase using specific antibody could significantly suppress the *S. eriocheiris* pathogenicity.

### Confirmation of the Interaction of MrLGBP With SeEnolase

**Figure 5:**
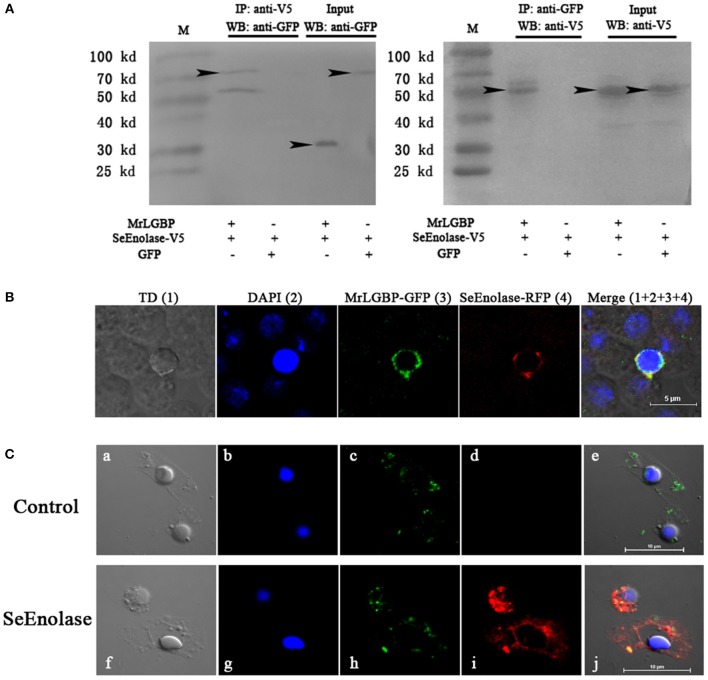
Interaction between MrLGBP and SeEnolase. **(A)** Co-immunoprecipitation demonstrating that GFP-tagged MrLGBP but not control GFP is co-precipitated with V5-tagged SeEnolase. Immunoprecipitation (IP) and western-blotting (WB) were performed using anti-V5 mouse antibody and anti-GFP rabbit antibodies, respectively. Input: western blotting of the input cell lysates before immunoprecipitation. Approximate molecular sizes: MrLGBP-GFP, ~68 kDa; SeEnolase-V5, ~50 kDa; GFP, ~28 kDa. **(B)** Subcellular localization of MrLGBP and SeEnolase. Recombinant plasmid of MrLGBP with GFP-tag and SeEnolase with RFP-tag were co-transfected into *Drosophila* S2 cells. MrLGBP and SeEnolase glowed green and red, respectively. Nuclei were stained with DAPI (blue). Bars = 5 μm. **(C)** The co-localization of MrLGBP and SeEnolase in prawn hemocytes analyzed by confocal microscopy. Hemocytes were incubated with SeEnolase protein for 1 h. For the control experiment, SeEnolase protein was not included. Hemocytes were incubated with mouse anti-MrLGBP and rabbit anti-SeEnolase as primary antibody, respectively. MrLGBP and SeEnolase stained by Alexa Flour 488 goat anti-Mouse IgG (green) and PE-labeled Goat Anti-Rabbit IgG (red). Hemocytes nuclei were stained with DAPI (blue). Bars = 10 μm. Hemocytes were visualized by bright field (a,f), blue fluorescence only (b,g), red fluorescence only (c,h), green fluorescence only (d,i), and by bright field merge with all fluorescent molecules (e,j) in both the control and SeEnolase group.

The expression of respective constructs was detected a strong co-precipitation of SeEnolase-V5 with MrLGBP-GFP (Figure 5A). In contrast, SeEnolase-V5 did not precipitate with control GFP. These data demonstrate a molecular interaction between MrLGBP and SeEnolase. Recombinant MrLGBP and SeEnolase were assessed in cultured *Drosophila* S2 cells using a confocal laser scanning microscope. MrLGBP and SeEnolase were found to co-localize in *Drosophila* S2 cells (Figure 5B). Both co-immunoprecipitation and co-localization demonstrated that SeEnolase binds MrLGBP directly in *Drosophila* S2 cells. As shown in Figure 5C, MrLGBP protein with green fluorescence and SeEnolase protein with red fluorescence could overlap to produce yellow fluorescence. These results showed that the interaction of MrLGBP and SeEnolase was present not only in *Drosophila* S2 cells but also in *M. rosenbergii* hemocytes.

### Overexpression of MrLGBP Promote *Drosophila* S2 Cells to Resist *S. eriocheiris* Infection

**Figure 6:**
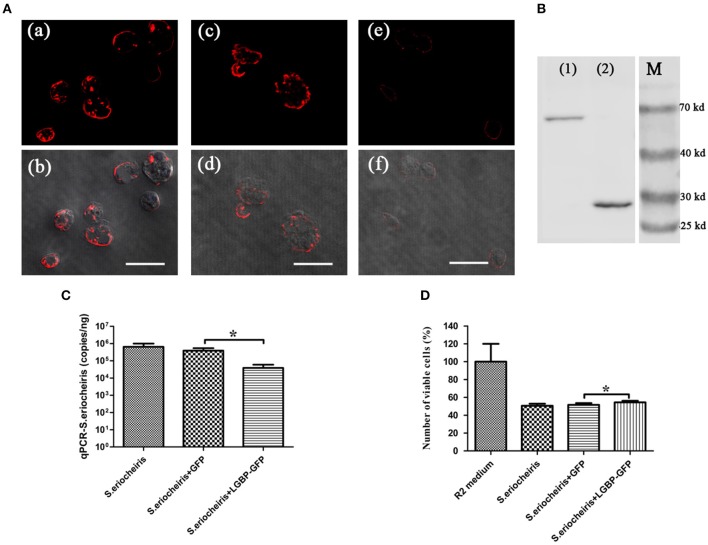
Overexpression of MrLGBP promote *Drosophila* S2 cells to resist *S. eriocheiris* infection. **(A)** Immunocytochemistry analysis *S. eriocheiris* quantity in *Drosophila* S2 cells. *Drosophila* S2 cells were transfected with pAc5.1-MrLGBP-GFP or pAc5.1-GFP plasmids. *Drosophila* S2 cells were infected with *S. eriocheiris* for 48 h. Then, the cells were incubated with anti-*S. eriocheiris* (primary antibody) and PE-labeled Goat anti-Rabbit IgG (second antibody, red), and examined using a confocal laser scanning microscope. (a,b) Represent the *S. eriocheiris* only group: (a), red fluorescence only, (b), the bright field merge with red fluorescence, (c,d) represents the *S. eriocheiris* + GFP group: (c), red fluorescence only, (d), bright field merge with red fluorescence, (e,f), represent the *S. eriocheiris* + LGBP-GFP group: (e), red fluorescence only, (f), bright field merge with red fluorescence, Bars, 10 μm. **(B)** Western blot of MrLGBP expression in *Drosophila* S2 cells. *Drosophila* S2 cells were transfected with pAc5.1-MrLGBP-GFP or pAc5.1-GFP plasmids. Efficiency of MrLGBP overexpression in *Drosophila* S2 cells, as detected by western blotting with anti-GFP antibody. (1), experimental group; (2), control group; (M), protein marker. Approximate molecular sizes: MrLGBP, ~68 kDa; GFP, ~28 kDa. **(C)** Real-time PCR analysis of copy number for *S. eriocheiris* in *Drosophila* S2 cells. *Drosophila* S2 cells were transfected with pAc5.1-MrLGBP-GFP or pAc5.1-GFP plasmids. *Drosophila* S2 cells were infected with *S. eriocheiris* for 48 h. *S. eriocheiris* quantity in *Drosophila* S2 cells. Vertical bars depict the mean ± S.E (*n* = 9). **(D)** Cell viability by CCK-8 assay. *Drosophila* S2 cells were transfected with pAc5.1-MrLGBP-GFP or pAc5.1-GFP plasmids. *Drosophila* S2 cells were infected with *S. eriocheiris* for 48 h. Relative cell viability was tested using CCK-8 assay. Cells used for different treatments are shown on the abscissa and relative cell viability rate on the ordinate. The assay was repeated three times. Vertical bars depict the mean ± S.E (*n* = 36). Significant difference *p < 0.05.

To assess the role of MrLGBP protein on *S. eriocheiris* infection, *Drosophila* S2 cells were transfected with a pAc5.1-MrLGBP-GFP plasmid and then infected with *S. eriocheiris*. Results showed that the number of *S. eriocheiris* was decreased in the *S. eriocheiris* + LGBP-GFP group compared to the *S. eriocheiris* + GFP group and the *S. eriocheiris* only group (Figure 6A). The result of western blot shown that the expression of MrLGBP, GFP-fusion MrLGBP and the GFP-tag were successful (Figure 6B). Real-time PCR results showed that the copy number of *S. eriocheiris* was 38,869/ng total DNA in the *S. eriocheiris* + LGBP-GFP group at 48 h post infection, whereas 389,311/ng and 646,956/ng total DNA was found in the *S. eriocheiris* + GFP group and in the *S. eriocheiris* only group, respectively (Figure 6C). Based on the CCK-8 assay, relative cell viability of the LGBP-GFP + *S. eriocheiris* group was significantly higher (*p* < 0.05) than that in the *S. eriocheiris* + GFP and *S. eriocheiris* only groups at 48 h post *S. eriocheiris* infection. CCK-8 test results showed that relative cell viability was 100% in the R2 medium group, whereas 50.57, 51.65, and 54.37% viability was found in the *S. eriocheiris* only group, the *S. eriocheiris* + GFP group, and the LGBP-GFP + *S. eriocheiris* group, respectively (Figure 6D). In a word, the overexpression of MrLGBP could help *Drosophila* S2 cells to resist *S. eriocheiris* infection.

### *MrLGBP* Silencing Reduced the Ability of *M. rosenbergii* Resistance to *S. eriocheiris*

**Figure 7:**
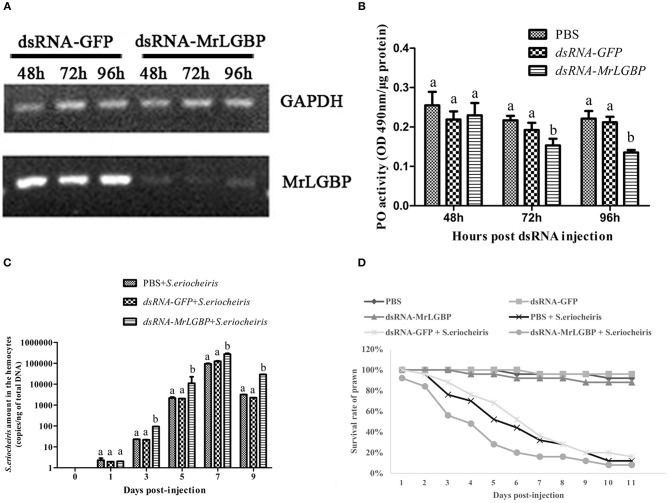
*MrLGBP* silencing reduced the ability of *M. rosenbergii* resistance to *S. eriocheiris*. **(A)** Efficiency of *MrLGBP* silencing in hemocytes, as detected by semi-quantitative PCR. **(B)** The phenol oxidase (PO) activity in hemocytes was determined after *MrLGBP* silencing. Differences between each group were analyzed using one-way ANOVA. Different letters indicate statistical significance (*p* < 0.05) and the same letter indicate no statistical difference (*p* > 0.05). **(C)** The quantification of *S. eriocheiris* copies in hemocytes from the three groups detected by real-time PCR at 1, 3, 5, 7, and 9 days, respectively. Statistical significance was determined by Student's *t*-test. Different letters indicate statistical significance (*p* < 0.05) and the same letter indicate no statistical difference (*p* > 0.05). **(D)** The survival rate of *MrLGBP* silencing prawn infected with *S. eriocheiris*. Prawn were divided six groups (50 prawn in each group). The prawns of the dsRNA-LGBP group and dsRNA-LGBP + *S. eriocheiris* group were injected with *MrLGBP* dsRNA. The prawns of the dsRNA-GFP group and the dsRNA-GFP + *S. eriocheiris* group were injected with GFP dsRNA. The prawns of the PBS group and PBS + *S. eriocheiris* group were injected with PBS. After 24 h, the prawns were injected again with the same amount dsRNA or PBS. Forty-eight hours after the first injection, the prawns of the PBS + *S. eriocheiris* group, dsRNA-GFP + *S. eriocheiris* group, and dsRNA-LGBP + *S. eriocheiris* group received an injection of *S. eriocheiris*. The cumulative mortality of prawns was recorded daily.

In order to assess *MrLGBP* function during the prawn immune response, *MrLGBP* was silenced using RNAi during pathogen infection. The data show (Figure 7A) that the *MrLGBP* transcription was declined dramatically in the dsRNA-MrLGBP group compared to the dsRNA-GFP group, and maintained for 96 h after MrLGBP dsRNA inoculation. Therefore, the interference assay for *MrLGBP* was high efficiency. After *MrLGBP* interference, *M. rosenbergii* hemocyte PO activity was remarkably lower from 72 to 96 h (Figure 7B). The results suggested that *MrLGBP* silencing might reduce the innate immunity of prawns.

The change in *S. eriocheiris* copies in *M. rosenbergii* hemocytes was measured by real-time PCR (Figure 7C). Meanwhile, the number of died prawns during this experiment was recorded in Table S5. The copy number of *S. eriocheiris* in the dsRNA-LGBP + *S. eriocheiris* group was significantly increased in the hemocytes from 3 to 9 days compared to the dsRNA-GFP + *S. eriocheiris* group (*p* < 0.05). The survival rate of *MrLGBP* dsRNA injected prawns was decreased compared with the dsRNA-GFP + *S. eriocheiris* group (Figure 7D). The cumulative survival rate of the dsRNA-LGBP + *S. eriocheiris* group was 8% at 11 day, compared to the dsRNA-GFP + *S. eriocheiris* group and PBS + *S. eriocheiris* group, which was 28 and 32%, respectively. These results showed that *MrLGBP* interference reduced the ability of *M. rosenbergii* resistance to *S. eriocheiris*.

### SeEnolase Induced Immune Responses and Improved the Ability of *M. rosenbergii* Resistance to *S. eriocheiris*

**Figure 8:**
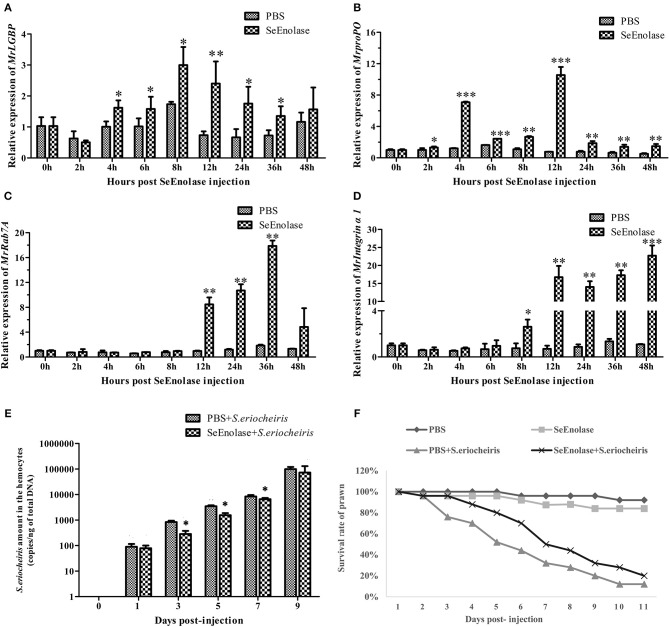
SeEnolase induced immune responses and improved the ability of *M. rosenbergii* resistance to *S. eriocheiris*. **(A)** Transcription levels of *MrLGBP* in hemocytes after SeEnolase stimulation. Prawns were injected with SeEolase protein as an experimental group and injected with PBS as control group. The hemocytes were sampled from every five individuals at 0, 2, 4, 6, 8, 12, 24, 36, and 48 h post-injection. Transcription levels of *MrLGBP* in hemocytes were analyzed by QRT-PCR. GAPDH was used as a reference gene. Statistical significance was determined by Student's *t*-test. Significant differences are indicated: *p < 0.05 and **p < 0.01. **(B)** QRT-PCR analysis of *MrproPO* in prawn hemocytes after SeEnolase stimulation. Statistical significance was determined by Student's *t*-test. Significant differences are indicated: *p < 0.05, **p < 0.01, and ***p < 0.001. **(C)** QRT-PCR analysis of *MrRab7A* in prawn hemocytes after SeEnolase stimulation. Statistical significance was determined by Student's *t*-test. Significant differences are indicated: **p < 0.01. **(D)** QRT-PCR analysis of *Mrintegrin* α*1* in prawn hemocytes after SeEnolase stimulation. Statistical significance was determined by Student's *t*-test. Significant differences are indicated: *p < 0.05, **p < 0.01, and ***p < 0.001. **(E)** the quantification of *S. eriocheiris* copies in hemocytes from the three groups detected by real-time PCR at 1, 3, 5, 7, and 9 days, respectively. Statistical significance was determined by Student's *t*-test. Significant differences are indicated: *p < 0.05. **(F)** The survival rate of SeEnolase stimulation prawn infected with *S. eriocheiris*. Prawns were injected with SeEnolase protein in SeEnolase + *S. eriocheiris* group. For PBS + *S. eriocheiris* group, prawns were injected with PBS. After 12 h stimulation, the prawns were received an injection of *S. eriocheiris*. Two another groups were injected with the SeEnolase or PBS only. The cumulative mortality of prawns was recorded daily.

The expression of immune genes mRNA after SeEnolase protein stimulation in hemocytes was detected by qRT-PCR. The *MrLGBP* mRNA in the SeEnolase stimulation group was significantly up-regulated (*p* < 0.05) from 4 to 36 h (Figure 8A). And, compared with the PBS group, the expression of gene *MrprpPO* in the SeEnolase injected group was remarkably up-regulated from 2 to 48 h and demonstrated the highest expression at 12 h (Figure 8B). In addition, *MrRab7A* and *Mrintegrin* α*1* genes were also obviously up-regulated compared with the control group from 12 to 36 h (Figure 8C) and 8 to 48 h (Figure 8D), respectively. These results indicated that the SeEnolase stimulation triggered the prawn innate immune response.

After being stimulated for another 12 h, the prawns were injected with *S. eriocheiris*. As shown in Figure 8E, the copy number of *S. eriocheiris* in the SeEnolase + *S. eriocheiris* group was significantly decreased in the hemocytes from 3 to 7 days compared to the PBS + *S. eriocheiris* group (*p* < 0.05). Meanwhile, the number of live prawns during this experiment was recorded in Table S6. The survival rate of the SeEnolase + *S. eriocheiris* group was increased comparison with the PBS + *S. eriocheiris* group (Figure 8F). The cumulative survival rate of the SeEnolase + *S. eriocheiris* group was 38% at 8 days, compared to the PBS + *S. eriocheiris* group, which was 8%. At the same time, cumulative survival rate of SeEnolase group and PBS group was 85 and 88%, respectively. The results showed that SeEnolase induced immune responses and enhanced the ability of *M. rosenbergii* resistance to *S. eriocheiris*.

## Discussion

Spiroplasma belongs to a mollicute species that lacks cell wall and does not produce external toxins or endotoxins (3). Therefore, proteins that mediate the interaction of spiroplasma with host cells likely play an important role in pathogenesis. Identification of those interactive proteins will provide for a greater understanding of host-pathogen relationships.

Using a far western blotting assay, Killiny et al. (36) demonstrated spiroplasma proteins to have an affinity for seven leafhopper proteins. A leafhopper protein overlay assay on an *S. citri* protein blot showed that spiralin, which is the most abundant membrane protein of *S. citri*, displayed insect protein-binding activity. In 2010, five significant binding activities among *S. citri* proteins and insect host proteins were identified in salivary glands using an *in vitro* protein overlay assay (21) that identified actin as one of the insect binding proteins. An *S. citri* actin-binding protein of 44 kDa was isolated by affinity chromatography and identified as PGK. Competitive spiroplasma attachment and internalization assays demonstrated that PGK-FL5-actin interaction is required for the internalization of *S. citri* (16). In their crustacean hosts, an essential step in the pathogenic spiroplasma's life cycle is hemocyte invasion. In this investigation, six potential receptors were identified, including Ran, LGBP, beta-Actin, proPO, beta tubulin, and alpha-tubulin. These six proteins can be classified into three groups, a cytoskeleton group including beta-Actin, beta tubulin, and alpha-tubulin; an innate immunity group including LGBP and proPO and a signal transduction group including Ran.

As previously described, bacteria binding analysis suggested that the *Fenneropenaeus chinensis* LGBP (Fc-LGBP) protein was able to strongly bind to Gram-negative bacteria, with little or no binding to Gram-positive bacteria or yeast (37). Herein, the *in vitro* spiroplasma binding assay and confocal analysis confirmed that MrLGBP also bound spiroplasma, though *S. eriocheiris* belongs to a mollicute species that lacks a cell wall. Therefore, our point is that MrLGBP could not only bind Gram-negative bacteria, but also the cell wall-less bacteria. In addition, Fc-LGBP hemocyte membrane localization (37) was confirmed by immunohistochemistry as similar as our result. This suggested that transmembrane MrLGBP protein might have a function of recognizing invading microorganisms.

Ligand proteins of MrLGBP were identified as SeEnolase, TK, ALDH, and DNA-directed RNA polymerase subunit beta. In competitive assays, recombinant proteins, SeEnolase, TK, and ALDH were added to *M. rosenbergii* primary hemocyte cultures prior to infection with spiroplasma. Significant difference was found with SeEnolase but little or no difference for TK or ALDH. Invasion by *S. eriocheiris* was decreased after competitive inhibition by SeEnolase. Enolase has been reported to play a role as plasminogen receptor on the surface of several pathogenic bacteria (38, 39), fungi (40), and protozoa (41–43). For example, in *Vibrio parahaemolyticus*, the glycolytic enzyme enolase, a membrane associated protein located on the cell surface was found to bind plasminogen (28). Similar results were obtained with *S. pneumoniae* (18) and *M. fermentans* (17). And, enolases have raised interest as inducers of protective immunity and potential vaccine candidates in two tapeworm infections in recent years (44, 45). In this study, a portion of SeEnolase was located on *S. eriocheiris'* membrane, where SeEnolase could directly bind to combine with MrLGBP *in vitro*. The result suggests that *S. eriocheiris* adhesion to *M. rosenbergii* hemocytes may be due to interaction between SeEnolase and MrLGBP. From further investigation, the virulence ability of *S. eriocheiris* was effectively reduced by anti-SeEnolase serum neutralization assay. These results suggest that the loss of SeEnolase function reduces the ability of *S. eriocheiris* adhesion to host cells. In other words, SeEnolase plays an important role in the process of *S. eriocheiris* invasion into *M. rosenbergii* hemocytes.

Pattern recognition proteins (PRPs), including LGBP and lipopolysaccharides, have an ability to bind to pathogen-associated molecular patterns, on the surface of microorganisms, triggering cellular responses to that resist penetration of various pathogens (46). And in general, subsequent to recognition, LGBP induced a series of immune responses including encapsulation, phagocytosis, and the activation of the prophenoloxidase (proPO) system (6, 47) in invertebrates (46). Over-expression of MrLGBP decreased invasion of *S. eriocheiris*, and increased cellular proliferation. MrLGBP interaction with *S. eriocheiris* activates the proPO system, which increases the cellular immune response. Meanwhile, RNAi depletion of *MrLGBP* significantly reduced *M. rosenbergii* hemocyte PO activity and prawn survival rate, increased *S. eriocheiris* copies at same time. These results suggest that silencing of *MrLGBP* increased *M. rosenbergii* sensitivity to *S. eriocheiris*. In a word, MrLGBP was involved in the regulation of *S. eriocheiris* invasion into to *M. rosenbergii* hemocytes. Herein, MrLGBP, a hemocyte receptor protein, was demonstrated to bind spiroplasma by direct interaction with SeEnolase. Therefore, SeEnolase stimulation might induce the prawn innate immune responses. It is well-known that LGBP and prpPO were important components of proPO system (6). In addition, Rab and integrin proteins could regulate invertebrate hemocytic phagocytosis (48, 49) and encapsulation (50, 51), respectively. Our research showed that the transcription of genes *MrLGBP, MrprpPO, MrRab7A*, and *Mrintegrin* α*1* were significantly up-regulated after SeEnolase stimulation, which suggested that prawn immune responses, including proPO system, phagocytosis and encapsulation were activated by SeEnolase. Crustaceans could use the innate immune to eliminate pathogens through activation of immune systems by LGBP. In our study, it was found that the activation of three immune systems by enolase stimulation led to an increase in resistibility against *S. eriocheiris* in *M. rosenbergii*. The evidence suggested that prawn immune system activation is due to SeEnolase binding to MrLGBP.

**Figure 9:**
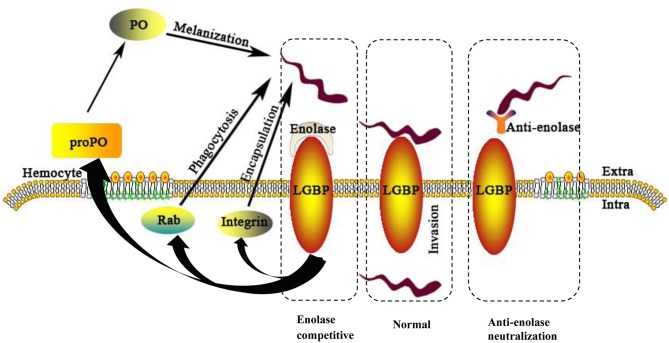
A schematic model of the *M. rosenbergii* hemocytes immune reaction against *S. eriocheiris* infected. For abbreviations and explanation see the text.

In conclusion, this investigation has identified interacting proteins between *S. eriocheiris* and *M. rosenbergii* hemocytes. Evidence showed *S. eriocheiris* SeEnolase, a surface-exposed protein, to promote pathogen-host interaction, involved in colonization and/or invasion of *S. eriocheiris* into *M. rosenbergii* hemocytes. In addition, MrLGBP, as a recognition protein, interacted with SeEnolase to defense the pathogen by activate prawn three innate immune systems (Figure 9). In summary, MrLGBP and SeEnolase involved in mediating *S. eriocheiris* invasion into *M. rosenbergii* hemocytes.

## Ethics Statement

The animal subjects used in the present study are freshwater prawn, which are invertebrates and are exempt from this requirement.

## Author Contributions

QM, MN, and YX designed experiments, analyzed experimental results, and wrote the manuscript. MY, JB, LH, WG, and WW conceived the idea, discussed data, and supervised this work.

### Conflict of Interest Statement

The authors declare that the research was conducted in the absence of any commercial or financial relationships that could be construed as a potential conflict of interest.

## Supplementary Material

Figure S1 Mass spectrometry analysis of receptor proteins. Red letters represent successful blast with NCBInr. **(A)** Ras-related nuclear protein (Ran), similar to *Marsupenaeus japonicus* Ran with a shared coverage of 18%; **(B)** lipopolysaccharide and beta-1,3-glucan binding protein (LGBP), similar to *M. rosenbergii* LGBP with a shared coverage of 8%; **(C)** beta-actin protein, similar to *Litopenaeus vannamei* beta-actin with a shared coverage of 37%; **(D)** prophenoloxidase protein (proPO), similar to *M. rosenbergii* proPO with a shared coverage of 13%; **(E)** beta tubulin protein, to *Penaeus monodon* beta tubulin with a shared coverage of 28%; **(F)** alpha-tubulin protein, similar to *Eriocheir sinensis* alpha-tubulin with a shared coverage of 24 %; **(G)** alpha-tubulin protein, similar to *Penaeus monodon* alpha-tubulin with a shared coverage of 10%.

Figure S2 Mass spectrometry of ligand proteins. Red letters represent successful blast with NCBInr. **(A)** 50 kDa band was identified as enolase, shared of 7%; **(B)** 70 kDa band identified as transketolase (TK), shared coverage of 8%; **(C)** 100 kDa band was identified as acetaldehyde dehydrogenase (ALDH), shared coverage of 6%; **(D)** 130 kDa band was identified as DNA-directed RNA polymerase subunit beta, shared coverage of 7%.

Figure S3 Nucleotide and deduced amino acid sequence of enolase, transketolase (TK), and acetaldehyde dehydrogenase (ALDH) from *S. eriocheiris*. Nucleotide mutagenesis of recombinant plasmids are boxed. **(A)** The SeEnolase N-terminal domain (I4–L134) is underlined and the C-terminal domain (A139–N448) shaded in gray. **(B)** The TK dehydrogenases domain is underlined (F87–G244) and the pyrimidine binding domain shaded in gray (Q345–E515). **(C)** The aldehyde dehydrogenases domain is underlined (M1–R279). Stop codons are indicated by asterisks (^*^).

Figure S4 Western blotting of *S. eriocheiris* protein and purified recombinant SeEnolase. M, protein marker. Lane 1, *S. eriocheiris*; lane 2, purified recombinant SeEnolase, anti-SeEnolase serum as primary antigen. Lane 3, *S. eriocheiris*; lane 4, purified recombinant SeEnolase, pre-immune serum as the primary antibody.

Table S1 Sequences of primers used in this study.

Table S2 Related outputs obtained from MASCOT analysis.

Table S3 Related outputs obtained from MASCOT analysis.

Table S4 The number of lived prawns.

Table S5 The number of lived prawns.

Table S6 The number of lived prawns.
