# Supplementary figures and images for: Spiroplasma eriocheiris Invasion Into Macrobrachium rosenbergii Hemocytes Is Mediated by Pathogen Enolase and Host Lipopolysaccharide and β-1, 3-Glucan Binding Protein
Source: Front Immunol. 2019 Aug 8;10:1852. doi: 10.3389/fimmu.2019.01852 (PMC6694788; doi:10.3389/fimmu.2019.01852)

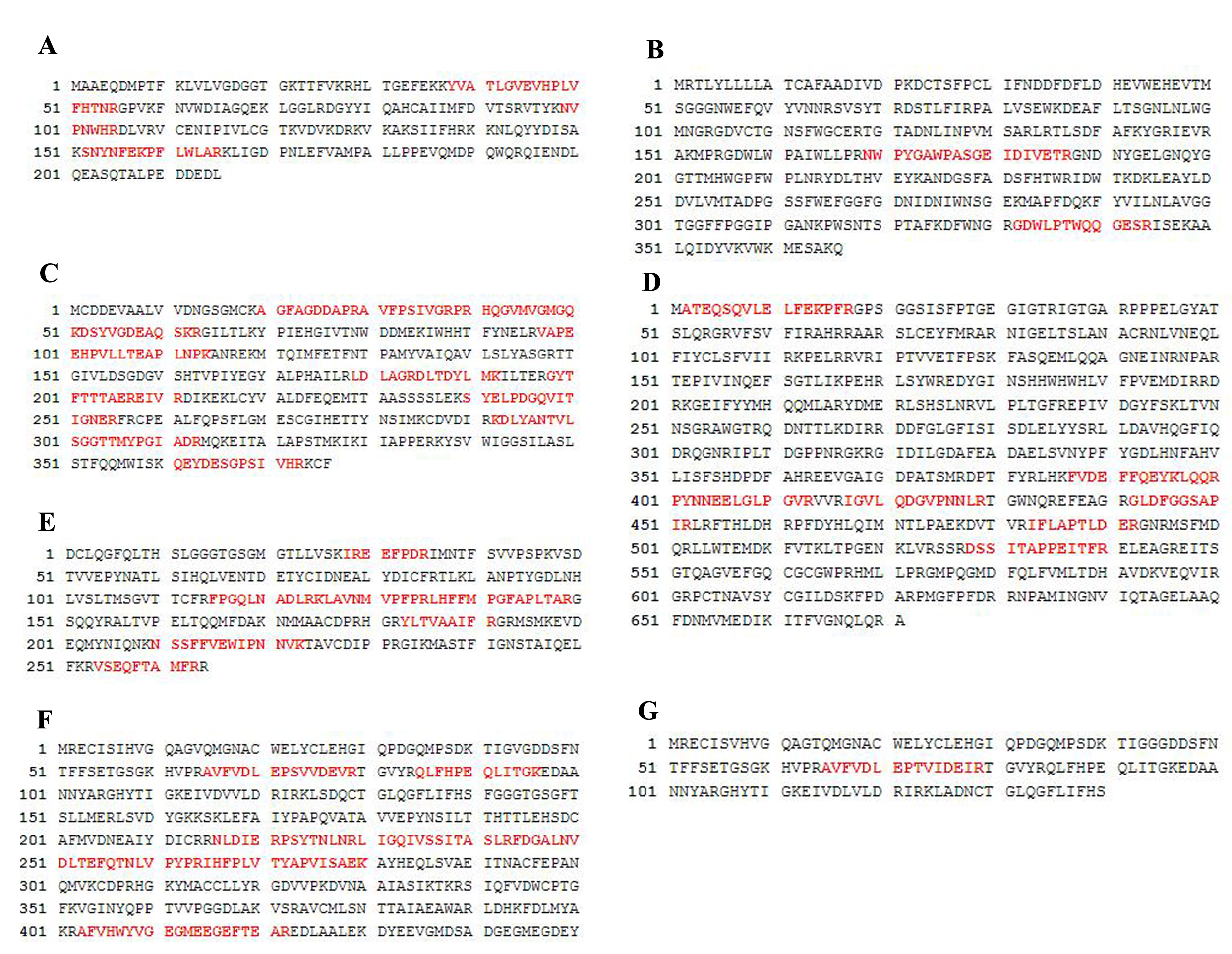

Supplement: Figure S1 — Mass spectrometry analysis of receptor proteins. Red letters represent successful blast with NCBInr. (A) Ras-related nuclear protein (Ran), similar to Marsupenaeus japonicus Ran with a shared coverage of 18%; (B) lipopolysaccharide and beta-1,3-glucan binding protein (LGBP), similar to M. rosenbergii LGBP with a shared coverage of 8%; (C) beta-actin protein, similar to Litopenaeus vannamei beta-actin with a shared coverage of 37%; (D) prophenoloxidase protein (proPO), similar to M. rosenbergii proPO with a shared coverage of 13%; (E) beta tubulin protein, to Penaeus monodon beta tubulin with a shared coverage of 28%; (F) alpha-tubulin protein, similar to Eriocheir sinensis alpha-tubulin with a shared coverage of 24 %; (G) alpha-tubulin protein, similar to Penaeus monodon alpha-tubulin with a shared coverage of 10%. [file Image_1.JPEG]

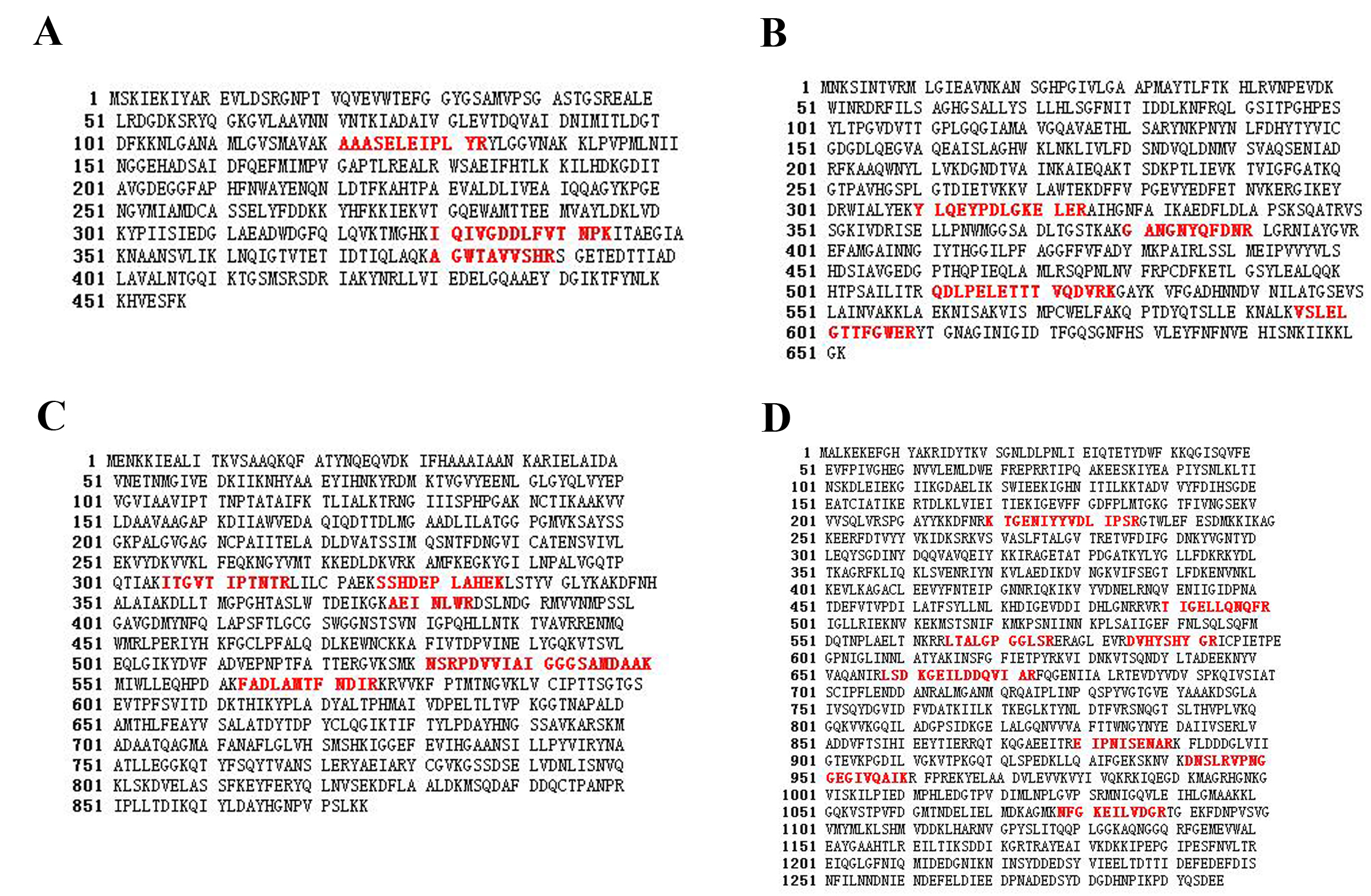

Supplement: Figure S2 — Mass spectrometry of ligand proteins. Red letters represent successful blast with NCBInr. (A) 50 kDa band was identified as enolase, shared of 7%; (B) 70 kDa band identified as transketolase (TK), shared coverage of 8%; (C) 100 kDa band was identified as acetaldehyde dehydrogenase (ALDH), shared coverage of 6%; (D) 130 kDa band was identified as DNA-directed RNA polymerase subunit beta, shared coverage of 7%. [file Image_2.JPEG]

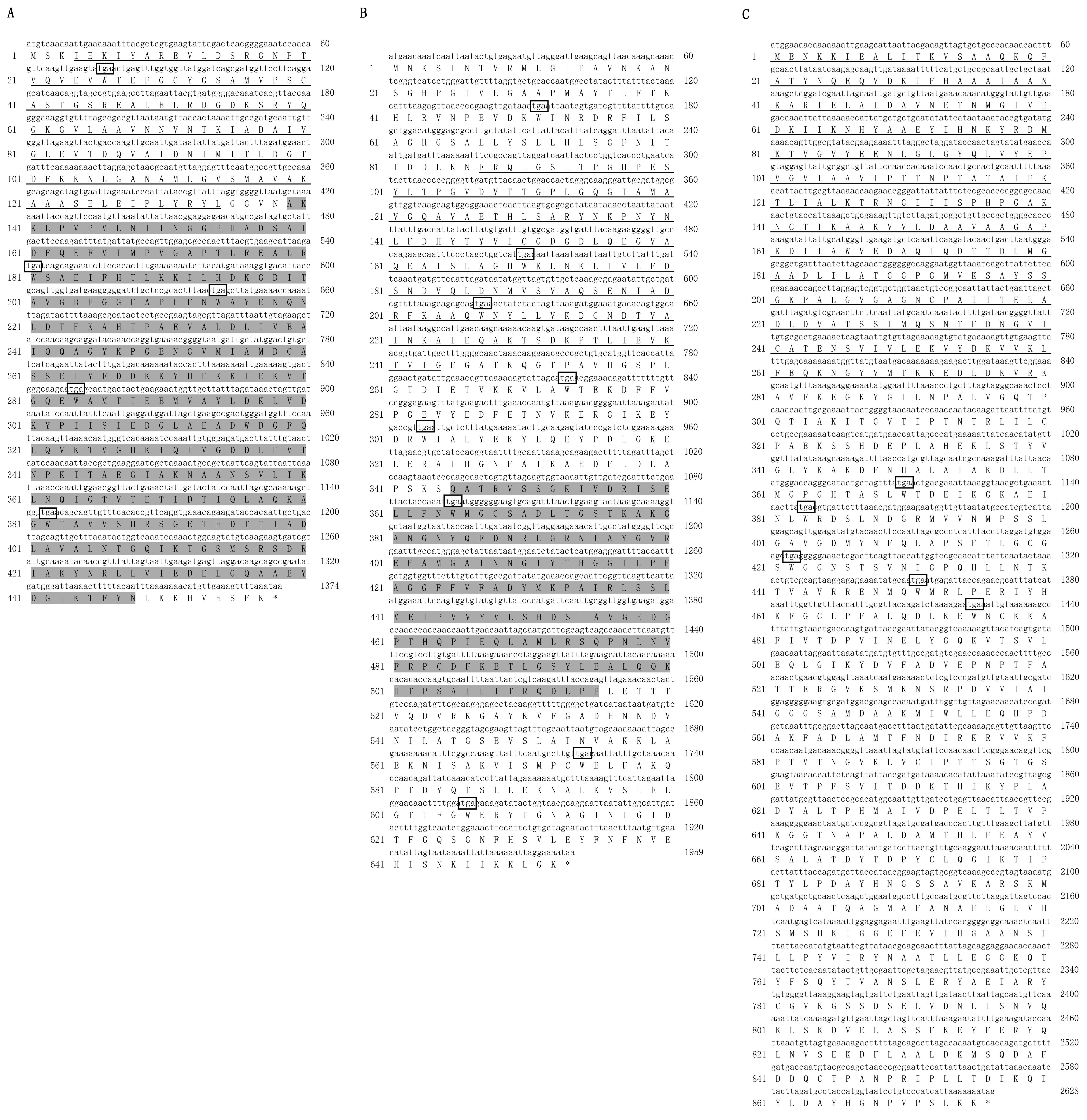

Supplement: Figure S3 — Nucleotide and deduced amino acid sequence of enolase, transketolase (TK), and acetaldehyde dehydrogenase (ALDH) from S. eriocheiris. Nucleotide mutagenesis of recombinant plasmids are boxed. (A) The SeEnolase N-terminal domain (I4–L134) is underlined and the C-terminal domain (A139–N448) shaded in gray. (B) The TK dehydrogenases domain is underlined (F87–G244) and the pyrimidine binding domain shaded in gray (Q345–E515). (C) The aldehyde dehydrogenases domain is underlined (M1–R279). Stop codons are indicated by asterisks (*). [file Image_3.jpg]

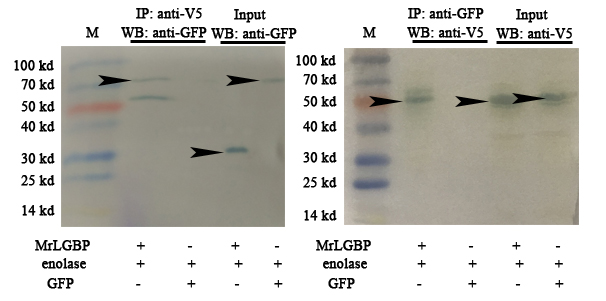

Supplement: Figure S4 — Western blotting of S. eriocheiris protein and purified recombinant SeEnolase. M, protein marker. Lane 1, S. eriocheiris; lane 2, purified recombinant SeEnolase, anti-SeEnolase serum as primary antigen. Lane 3, S. eriocheiris; lane 4, purified recombinant SeEnolase, pre-immune serum as the primary antibody. [file Image_4.JPEG]
