# Supplementary material for: Spiroplasma eriocheiris Invasion Into Macrobrachium rosenbergii Hemocytes Is Mediated by Pathogen Enolase and Host Lipopolysaccharide and β-1, 3-Glucan Binding Protein
Source: Front Immunol. 2019 Aug 8;10:1852. doi: 10.3389/fimmu.2019.01852 (PMC6694788; doi:10.3389/fimmu.2019.01852)
Supplement: Table S1 — Sequences of primers used in this study. [file Table_1.DOCX]

**Table S1** Sequences of primers used in this study.

| **Primer** | **Sequence (5’–3’)** |
| --- | --- |
| MrLGBP-F | GCCGGATCCGCTGATATTGTCGATCCTAAGG |
| MrLGBP-R | ATGGCGGCCGCTTACTGTTTCGCGCTCTCCA |
| Spiro enolase-F | ATGTCAAAAATTGAAAAAATTTACG |
| Spiro enolase-R | TTATTTAAAACTTTCAACATGTTTTTT |
| Spiro enolase-MutaF1 | TCAAGTTGAAGTATGGACTGAGTTTGGTGG |
| Spiro enolase-MutaR1 | CCATACTTCAACTTGAACTGTTGGATTTCC |
| Spiro enolase-MutaF2 | TGAAGCATTAAGATGGTCAGCAGAAA |
| Spiro enolase-MutaR2 | CCATCTTAATGCTTCACGTAAAGTT |
| Spiro enolase-MutaF3 | TCCGCACTTTAACTGGGCTTATGAAAAC |
| Spiro enolase-MutaR3 | CCAGTTAAAGTGCGGAGCAAATCCCCCT |
| Spiro enolase-MutaF4 | TGACTGGGCAAGAATGGGCAATGACTAC |
| Spiro enolase-MutaR4 | CCATTCTTGCCCAGTCACTTTTTCAATT |
| Spiro enolase-MutaF5 | CGCAAAAAGCTGGGTGGACAGCAGTTGT |
| Spiro enolase-MutaR5 | CCACCCAGCTTTTTGCGCTAATTGGATA |
| Spiro TK-F | ATGAACAAATCAATTAATACTGTGAGAA |
| Spiro TK-R | TTATTTTCCTAATTTTTTAATAATTTTATTAC |
| Spiro TK-MutaF1 | CGAAGTTGATAAATGGATTAATCGTGATCG |
| Spiro TK-MutaR1 | CCATTTATCAACTTCGGGGTTAACTCTTAA |
| Spiro TK-MutaF2 | CCTAGCTGGTCATTGGAAATTAAATAAATT |
| Spiro TK-MutaR2 | CCAATGACCAGCTAGGGAAATTGCTTC |
| Spiro TK-MutaF3 | TAAAGCAGCGCAGTGGAACTATCTACTAGT |
| Spiro TK-MutaR3 | CCACTGCGCTGCTTTAAAACGATCA |
| Spiro TK-MutaF4 | AAAAGTATTAGCATGGACGGAAAAAGATTT |
| Spiro TK-MutaR4 | CCATGCTAATACTTTTTTAACTGTTTCAATA |
| Spiro TK-MutaF5 | AGAATATGACCGTTGGATTGCTCTTTATGA |
| Spiro TK-MutaR5 | CCAACGGTCATATTCTTTAATTCCCCGT |
| Spiro TK-MutaF6 | ATTACTACCAAATTGGATGGGGGGAAGT |
| Spiro TK-MutaR6 | CCAATTTGGTAGTAATTCAGAAATGCGATC |
| Spiro TK-MutaF7 | TTCAATGCCTTGTTGGGAATTATTTGCTA |
| Spiro TK-MutaR7 | CCAACAAGGCATTGAAATAACTTTGG |
| Spiro TK-MutaF8 | AACAACTTTTGGATGGGAAAGATATACTGG |
| Spiro TK-MutaR8 | CCATCCAAAAGTTGTTCCTAATTCTAATGA |
| Spiro ALDH-F | ATGGAAAACAAAAAAATTGAAGC |
| Spiro ALDH-R | CTATTTTTTTAATGATGGGACAGGAT |
| Spiro ALDH-MutaF1 | TACTGCTAGTTTATGGACTGACGAAATT |
| Spiro ALDH-MutaR1 | CCATAAACTAGCAGTATGCCCTGGT |
| Spiro ALDH-MutaF2 | TGAAATTAACTTATGGCGTGATTCTTTAAA |
| Spiro ALDH-MutaR2 | CCATAAGTTAATTTCAGCTTTACCTTTA |
| Spiro ALDH-MutaF3 | AGGATGTGGAAGCTGGGGGGGAAAC |
| Spiro ALDH-MutaR3 | CCAGCTTCCACATCCTAAGGTAAATGAGG |
| Spiro ALDH-MutaF3 | CTTAGGATGTGGAAGCTGGGGGGGAAACTC |
| Spiro ALDH-MutaR3 | CCAGCTTCCACATCCTAAGGTAAATGAGGG |
| Spiro ALDH-MutaF4 | AGAAAATATGCAATGGATGAGATTACCA |
| Spiro ALDH-MutaR4 | CCATTGCATATTTTCTCTCCTTACTGCG |
| Spiro ALDH-MutaF5 | AGATCTAAAAGAATGGAATTGTAAAAAAGC |
| Spiro ALDH-MutaR5 | CCATTCTTTTAGATCTTGTAACGCAAAT |
| pAc-MrLGBP-F | GGGGTACCAAAATGGCTGATATTGTCGATCCTAAGG |
| pAc-MrLGBP-R | TTGGGCCCCTTACTGTTTCGCGCTCTCCA |
| pAc-enolase-F | GGGGTACCAAAATGGCAAAAATTGAAAAAATTTACG |
| pAc-enolase-R | TTGGGCCCTTATTTAAAACTTTCAACATGTTTTTT |
| Se-QF | CGCAGACGGTTTAGCAAGTTTGGG  AGCACCGAACTTAGTCCGACAC |
| Se-QR | AGCACCGAACTTAGTCCGACAC |
| dsRNA-MrLGBP-F | GCGTAATACGACTCACTATAGGTCCTTTGCCGACAGCTTCCATAC |
| dsRNA-MrLGBP-R | GCGTAATACGACTCACTATAGGGGTTTCCAGCAATCATCCGTTCA |
| dsRNA-GFP-F | GCGTAATACGACTCACTATAGGTGGTCCCAATTCTCGTGGAAC |
| dsRNA-GFP-R | GCGTAATACGACTCACTATAGGCTTGAAGTTGACCTTGATGCC |
| GAPDH-qF | TGCCGCCCAGAACATCATT |
| GAPDH-qR | TCGTCTTCGGTGTAGCCCA |
| MrLGBP-qF | TCAGACCTGCTTTGGTGTCCGAA |
| MrLGBP-qR | GGTTAATGAGGTTATCGGCGGTG |
| MrproPO-qF | ACACTGAAGGACATAAGGCGAGAT |
| MrproPO-qR | AGTAGAGTTCCAAGTCGGAGATGCT |
| MrRab7A-qF | AGTGGTGTCACAGTAAAAACGAGGT |
| MrRab7A-qR | GCGTCTTGCTTTGTCTTGTTATCAT |
| Mrintegrin α1-qF | AGAAACGACGGCATTAGA |
| Mrintegrin α1-qR | ACAAGGACGGATACCAGG |
