# Supplementary material for: Spiroplasma eriocheiris Invasion Into Macrobrachium rosenbergii Hemocytes Is Mediated by Pathogen Enolase and Host Lipopolysaccharide and β-1, 3-Glucan Binding Protein
Source: Front Immunol. 2019 Aug 8;10:1852. doi: 10.3389/fimmu.2019.01852 (PMC6694788; doi:10.3389/fimmu.2019.01852)
Supplement: Table S2 — Related outputs obtained from MASCOT analysis. [file Table_2.DOCX]

| Blast result | Mascot score | cover percentage | number of matched peptides |
| --- | --- | --- | --- |
| Ras-related nuclear protein [*Marsupenaeus japonicus*] | 133 | 18% | 3 |
| lipopolysaccharide and beta-1,3-glucan binding protein [*Macrobrachium rosenbergii*] | 118 | 8% | 2 |
| prophenoloxidase [*Macrobrachium rosenbergii*] | 410 | 13% | 7 |
| beta tubulin [*Penaeus monodon*] | 435 | 28% | 7 |
| alpha-tubulin [*Eriocheir sinensis*] | 434 | 24% | 7 |
| alpha-tubulin [*Penaeus monodon*] | 66 | 10% | 1 |

**Table S2** Related outputs obtained from MASCOT analysis.
