# Supplementary material for: Spiroplasma eriocheiris Invasion Into Macrobrachium rosenbergii Hemocytes Is Mediated by Pathogen Enolase and Host Lipopolysaccharide and β-1, 3-Glucan Binding Protein
Source: Front Immunol. 2019 Aug 8;10:1852. doi: 10.3389/fimmu.2019.01852 (PMC6694788; doi:10.3389/fimmu.2019.01852)
Supplement: Table S3 — Related outputs obtained from MASCOT analysis. [file Table_3.DOCX]

| Blast result | Mascot score | cover percentage | number of matched peptides |
| --- | --- | --- | --- |
| Enolase [*Spiroplasma eriocheiris*] | 75 | 7% | 3 |
| Transketolase [*Spiroplasma eriocheiris*] | 72 | 8% | 4 |
| acetaldehyde dehydrogenase [*Spiroplasma eriocheiris*] | 128 | 6% | 5 |
| DNA-directed RNA polymerase subunit beta [*Spiroplasma eriocheiris*] | 54 | 7% | 8 |

**Table S3** Related outputs obtained from MASCOT analysis.
