# Supplementary material for: Spiroplasma eriocheiris Invasion Into Macrobrachium rosenbergii Hemocytes Is Mediated by Pathogen Enolase and Host Lipopolysaccharide and β-1, 3-Glucan Binding Protein
Source: Front Immunol. 2019 Aug 8;10:1852. doi: 10.3389/fimmu.2019.01852 (PMC6694788; doi:10.3389/fimmu.2019.01852)
Supplement: Table S4 — The number of lived prawns. [file Table_4.DOCX]

**Table S4** The number of lived prawns.

|  | 0d | 1d | 2d | 3d | 4d | 5d | 6d | 7d | 8d | 9d | 10d | 11d |
| --- | --- | --- | --- | --- | --- | --- | --- | --- | --- | --- | --- | --- |
| PBS | 0 | 50 | 50 | 50 | 50 | 50 | 48 | 48 | 48 | 48 | 46 | 46 |
| pre-immune serum | 0 | 50 | 50 | 48 | 48 | 48 | 46 | 46 | 44 | 44 | 44 | 42 |
| anti-enolase serum | 0 | 50 | 50 | 50 | 48 | 46 | 45 | 44 | 43 | 43 | 41 | 40 |
| PBS+*S.eriocheiris* | 0 | 50 | 48 | 38 | 35 | 26 | 22 | 16 | 14 | 10 | 6 | 6 |
| pre-immune serum +*S.eriocheiris* | 0 | 48 | 46 | 36 | 32 | 29 | 23 | 17 | 15 | 9 | 7 | 7 |
| anti-enolase serum+S.eriocheiris | 0 | 50 | 50 | 46 | 42 | 38 | 30 | 24 | 22 | 16 | 10 | 10 |
