# Supplementary material for: Spiroplasma eriocheiris Invasion Into Macrobrachium rosenbergii Hemocytes Is Mediated by Pathogen Enolase and Host Lipopolysaccharide and β-1, 3-Glucan Binding Protein
Source: Front Immunol. 2019 Aug 8;10:1852. doi: 10.3389/fimmu.2019.01852 (PMC6694788; doi:10.3389/fimmu.2019.01852)
Supplement: Table S5 — The number of lived prawns. [file Table_5.DOCX]

**Table S5** The number of lived prawns.

|  | 0 d | 1 d | 2 d | 3 d | 4 d | 5 d | 6 d | 7 d | 8 d | 9 d | 10 d | 11 d |
| --- | --- | --- | --- | --- | --- | --- | --- | --- | --- | --- | --- | --- |
| PBS | 50 | 50 | 50 | 50 | 50 | 50 | 48 | 48 | 48 | 48 | 46 | 46 |
| dsRNA-GFP | 50 | 50 | 50 | 50 | 50 | 50 | 50 | 48 | 48 | 48 | 48 | 48 |
| dsRNA-LGBP | 50 | 50 | 50 | 50 | 48 | 48 | 46 | 46 | 46 | 44 | 44 | 44 |
| PBS + *S.eriocheiris* | 50 | 50 | 48 | 38 | 35 | 26 | 22 | 16 | 14 | 10 | 6 | 6 |
| dsRNA-GFP + *S.eriocheiris* | 50 | 50 | 48 | 44 | 38 | 34 | 26 | 18 | 14 | 10 | 10 | 8 |
| dsRNA-LGBP + *S.eriocheiris* | 50 | 46 | 42 | 28 | 24 | 14 | 10 | 8 | 8 | 6 | 4 | 4 |
