# Supplementary material for: Spiroplasma eriocheiris Invasion Into Macrobrachium rosenbergii Hemocytes Is Mediated by Pathogen Enolase and Host Lipopolysaccharide and β-1, 3-Glucan Binding Protein
Source: Front Immunol. 2019 Aug 8;10:1852. doi: 10.3389/fimmu.2019.01852 (PMC6694788; doi:10.3389/fimmu.2019.01852)
Supplement: Table S6 — The number of lived prawns. [file Table_6.DOCX]

**Table S6** The number of lived prawns.

|  | 0d | 1d | 2d | 3d | 4d | 5d | 6d | 7d | 8d | 9d | 10d | 11d |
| --- | --- | --- | --- | --- | --- | --- | --- | --- | --- | --- | --- | --- |
| PBS | 50 | 50 | 50 | 50 | 50 | 50 | 48 | 48 | 48 | 48 | 46 | 46 |
| enolase | 50 | 50 | 48 | 48 | 48 | 48 | 46 | 44 | 44 | 42 | 42 | 42 |
| PBS+ *S.eriocheiris* | 50 | 50 | 48 | 38 | 35 | 26 | 22 | 16 | 14 | 10 | 6 | 6 |
| enoalse+ *S.eriocheiris* | 50 | 50 | 48 | 48 | 44 | 40 | 35 | 25 | 22 | 16 | 14 | 10 |
